# Supplementary material for: ROCKII inhibition promotes the maturation of human pancreatic beta-like cells
Source: Nat Commun. 2017 Aug 21;8:298. doi: 10.1038/s41467-017-00129-y (PMC5563509; doi:10.1038/s41467-017-00129-y)
Supplement: Supplementary file 1 — Supplementary Information [file 41467_2017_129_MOESM1_ESM.pdf]

File name: Supplementary Information
Description: Supplementary Figures and Supplementary Tables.

File name: Peer Review File
Description:

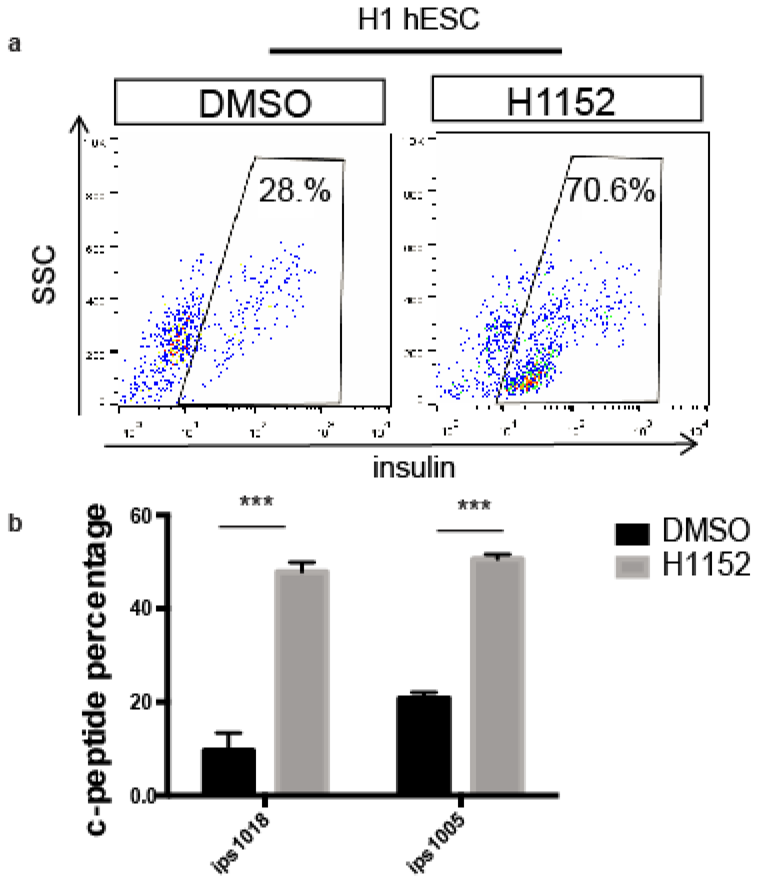

Supplementary Figure 1. The effect of H1152 on different hESC/iPSC lines.
Related with Figure 1. (a) Intracellular FCM to detect the expression of insulin in DMSO or H1152 treated H1-derived cells.
(b) Quantification of the percentage of c-peptide^{+} cells of DMSO or H1152 treated cells derived from 2 different iPSC lines. *p* values were calculated by unpaired two-tailed Student's t-test. ****p*<0.001. N=3-6 independent biological replicates. Error bar is SEM.

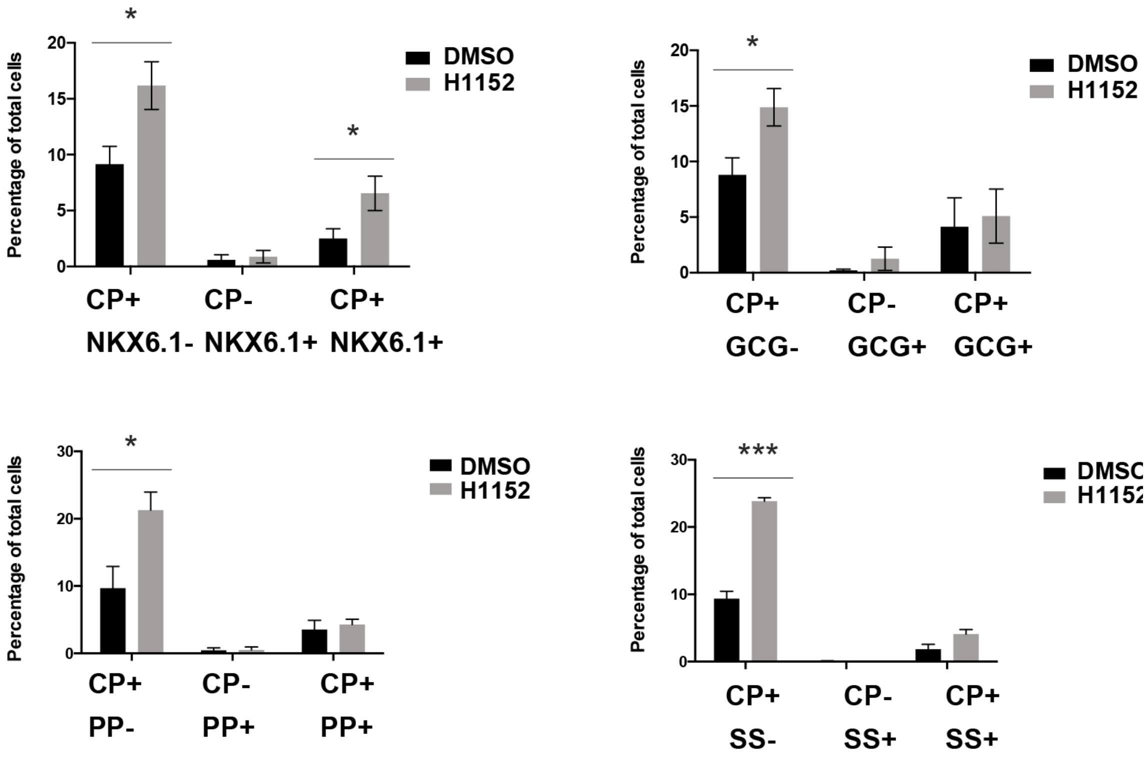

Supplementary Figure 2. H1152 promotes the maturation of hESC-derived glucose-responding cells. Related with Figure 2. Quantification of the intracellular FCM of H1152-treated vs DMSO-treated hESC-derived glucose-responding cells in Fig. 2d. *p* values were calculated by unpaired two-tailed Student's t-test. **p*<0.05, ***p*<0.01. N=3-6 independent biological replicates. Error bar is SEM.

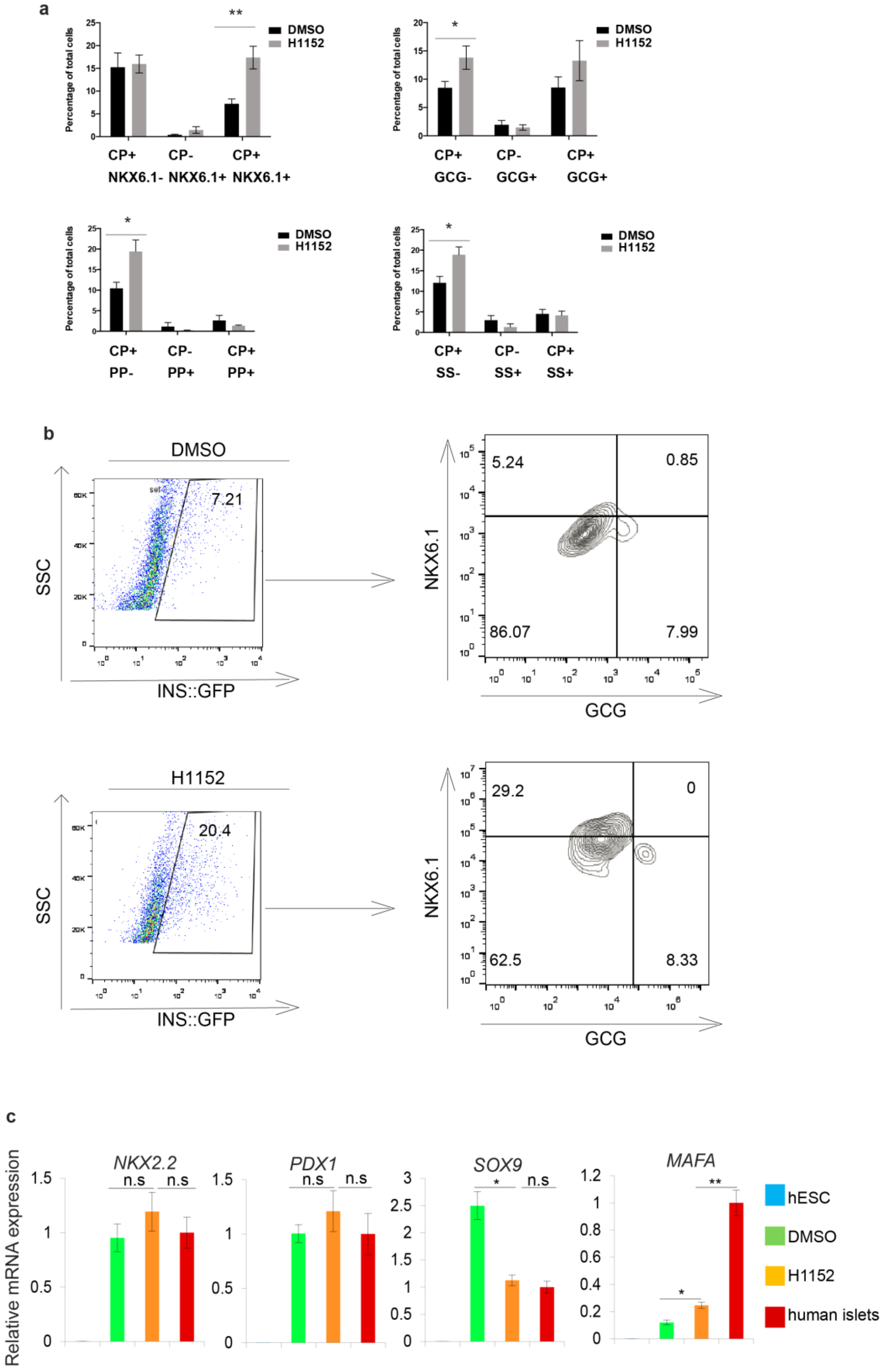
a
DMSO
H1152
Percentage of total cells
CP+ NKX6.1-
CP- NKX6.1+
CP+ NKX6.1+
CP+ GCG-
CP- GCG+
CP+ GCG+
CP+ PP-
CP- PP+
CP+ PP+
CP+ SS-
CP- SS+
CP+ SS+
b
DMSO
H1152
SSC
INS::GFP
NKX6.1
GCG
7.21
5.24
0.85
86.07
7.99
20.4
29.2
0
62.5
8.33
c
Relative mRNA expression
NKX2.2
PDX1
SOX9
MAFA
n.s
hESC
DMSO
H1152
human islets

Supplementary Figure 3. H1152 promotes the generation and maturation of hESC-derived glucose-responding cells in the presence of a distinct protocol. Related with Figure 2. (a) Intracellular FCM quantification for H1152-treated vs DMSO-treated hESC-derived glucose-responding cells in Fig. 4c. N=3-6 independent biological replicates (b) NKX6.1 and GCG staining of purified INS-GFP$^+$ cells in H1152- and DMSO- treated population. (c) qRT-PCR analysis of H1152-treated or DMSO-treated cells. Scale bar: 100 µm. The data were normalized to primary human islets. N=6-8 independent biological replicates. *p* values were calculated by unpaired two-tailed Student's t-test. *$p<0.05$, **$p<0.01$. Error bar is SEM.

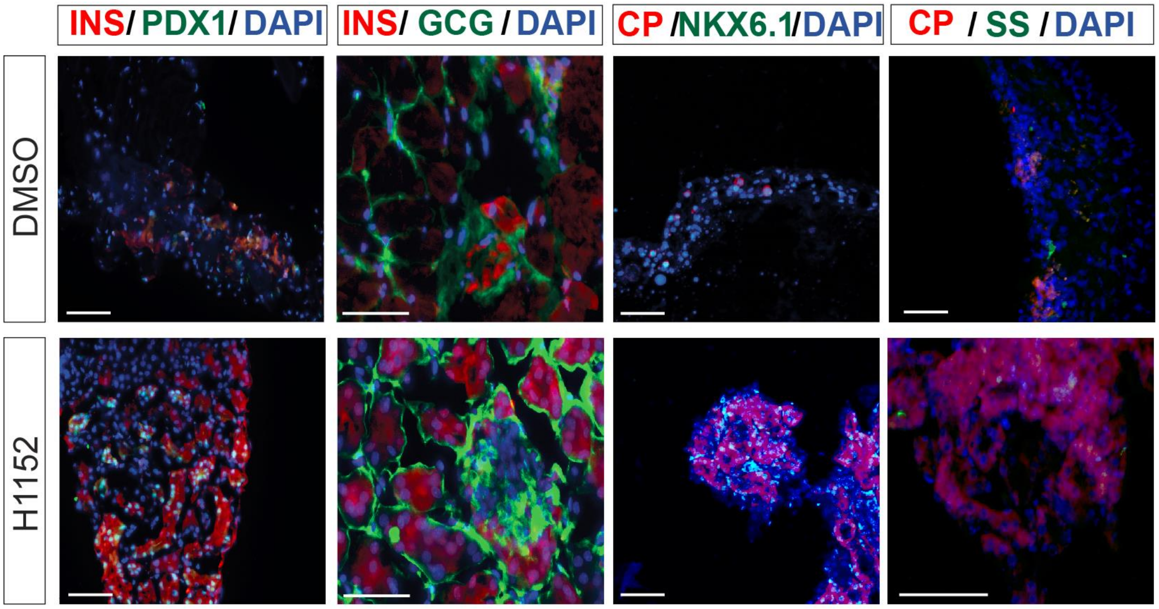

Supplementary Figure 4. Immunohistochemistry analysis of grafts of H1152-treated or DMSO-treated cells. Related with Figure 5. Scale bar: 100 µm. GCG: glucagon; SS: somatostatin; CP:c-peptide.

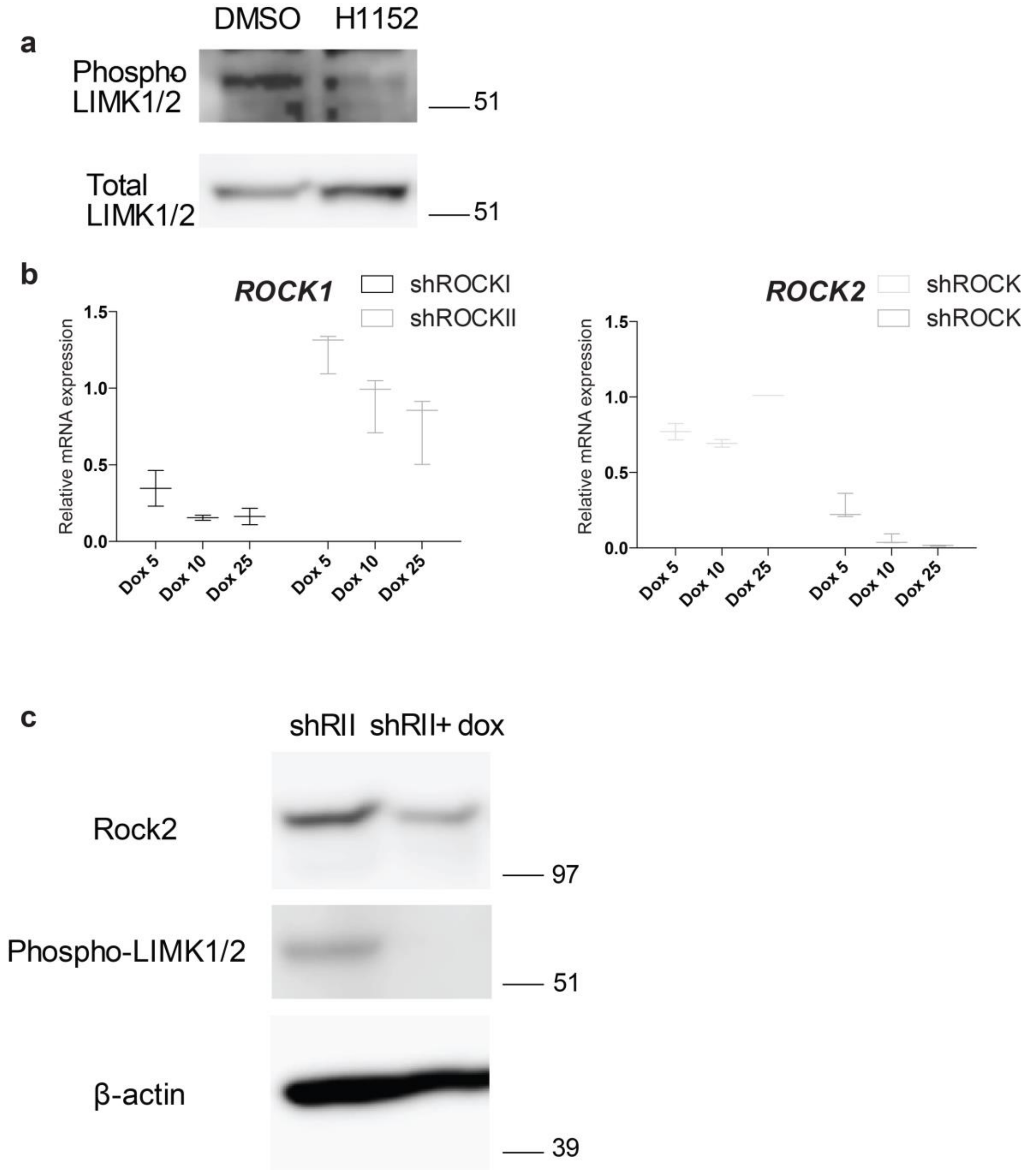

Supplementary Figure 5. ROCKII, but not ROCK I, inhibition promotes the generation and maturation of glucose-responding cells. Related with Figure 6. (a) Western blotting analysis of lysates from HUES8-derived cells at 48 hours after DMSO or H1152 (10 µM) treatment. (b) qRT-PCR to validate the knockdown efficiency in hESCs at 5, 10 or 25 µg/mL dox concentrations. N=3 independent biological replicates. Error bar is SEM. (c) Western blotting analysis of lysates from HUES8-derived cells at 48 hours after 10 µg/mL doxycycline treatment.

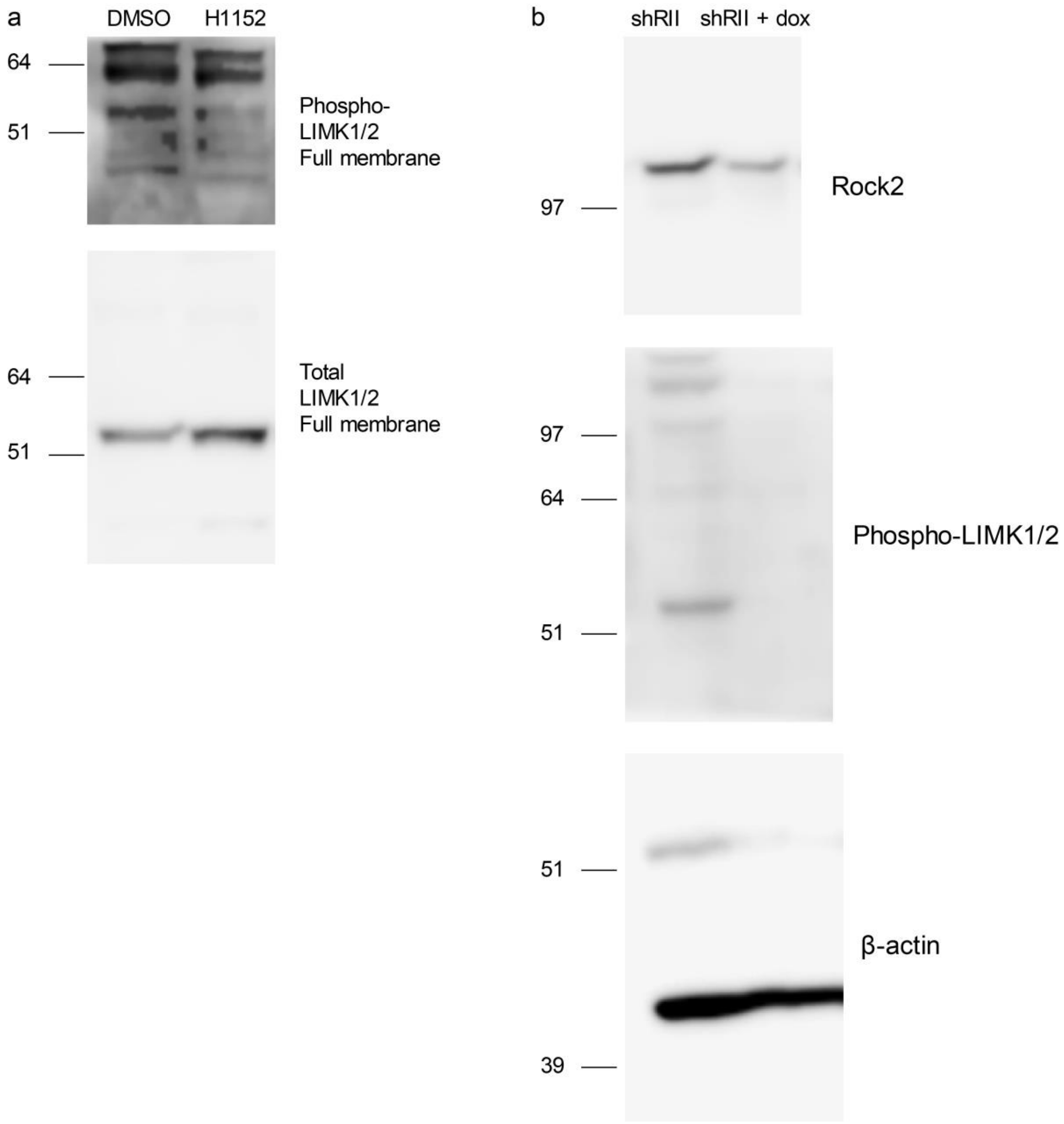

Supplementary Figure 6. Uncropped western blots of Supplementary Figure 5a (a) and Supplementary Figure 5c (b).

Supplementary Table 1. Related with Figure 1. Primary screening data

Category	Parameter	Description
Assay	Type of assay	Cell-based
	Target	Pancreatic beta cell generation and maturation
	Primary measurement	Percentage of insulin+ cells
	Key reagents	Insulin antibody
	Assay protocol	See the section of experimental procedures.
	Additional comments	
Library	Library size	More than 4000 compounds
	Library composition	FDA approved drugs, kinase inhibitors, signaling pathway regulators
	Source	Sigma LOPAC library, Microsource Spectrum library
	Additional comments	
Screen	Format	384-well plate
	Concentration(s) tested	10 μM and 1 μM compounds
	Plate controls	None
	Reagent/ compound dispensing system	Multidrop
	Detection instrument and software	Molecular Device ImageXpress^{Micro} Automated High Content Analysis System
	Assay validation/QC	Standard deviation of controls and background
	Correction factors	Z score=0.59
	Normalization	None
	Additional comments	
Post-HTS analysis	Hit criteria	The compounds that increase the percentage of INS^+ cells by three folds were picked as primary hits.
	Hit rate	About 0.2% (8/4000)
	Additional assay(s)	Intracelluar FACS to detect the percentage of insulin+ cells and c-peptide+ cells
	Confirmation of hit purity and structure	Compounds were repurchased from Tocris
	Additional comments	

Supplementary Table 2. Hit compounds from primary screening.

Compound name	Chemical structure	Efficacy Curve	Toxicity Curve
H1152	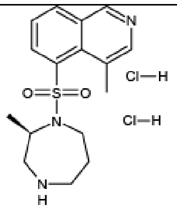	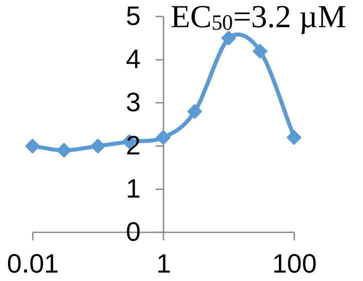	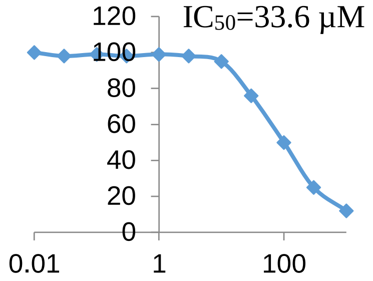
BWB70C	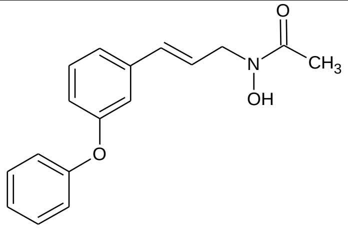	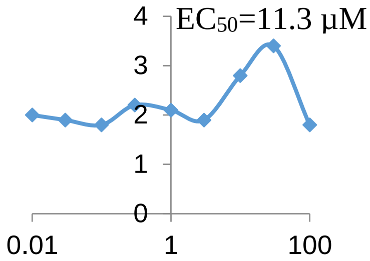	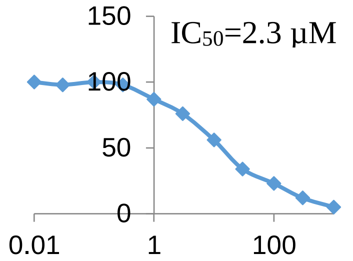
Chicago sky blue 6B	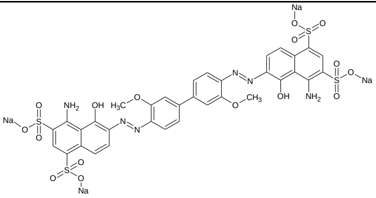	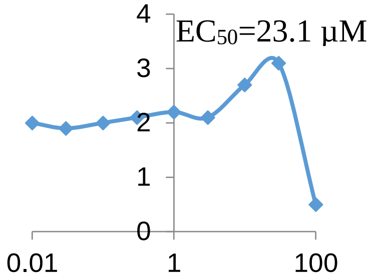	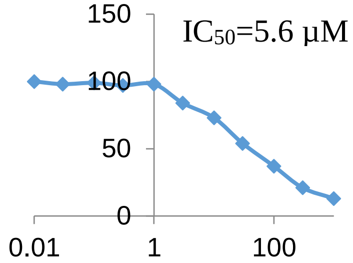
Genipin	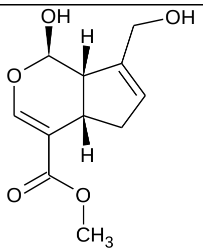	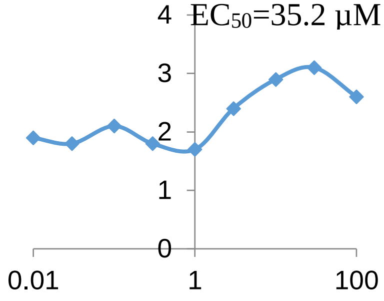	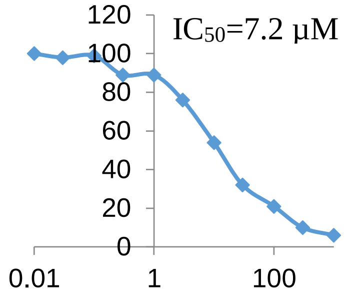
Y-27632	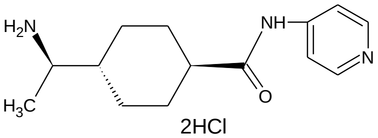	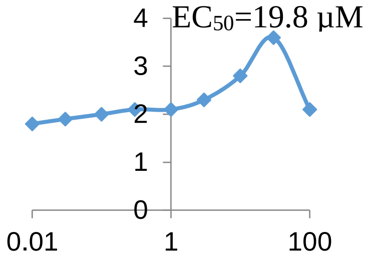	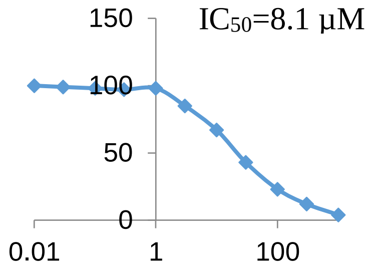

Supplementary Table 3. Related with Figure 4. Differentiation Protocol.

S1 (3 days)	1 Day	MCDB131<br>1.5g /L Sodium bicarbonate<br>10 mM glucose<br>0.5% Fatty acid free BSA	100 ng/Ml activin A<br>3 μM Chir99021
	1 Day	MCDB131<br>1.5 g/L Sodium bicarbonate<br>10 mM glucose<br>0.5% Fatty acid free BSA	100 ng/mL activin A<br>0.3 μM Chir99021
	1 Day	MCDB131<br>1.5g/L Sodium bicarbonate<br>10mM glucose<br>0.5% Fatty acid free BSA	100ng/mL activin A
S2 (2 days)	2 Days	MCDB131<br>1.5 g/L Sodium bicarbonate<br>10 mM glucose<br>0.5% Fatty acid free BSA	0.25 mM ascorbic acid<br>50 ng/mL FGF7
S3 (2 days)	2 Days	MCDB131<br>2.5 g/L Sodium bicarbonate<br>10 mM glucose<br>2% Fatty acid free BSA	0.25 mM ascorbic acid<br>50 ng/mL FGF7<br>0.25 μM SANT-1<br>1 μM retinoic acid<br>100 nM LDN193189<br>1:200 ITS-X<br>200 nM TPB
S4 (3 days)	3 Days	MCDB131<br>2.5 g/L Sodium bicarbonate<br>10 mM glucose<br>2% Fatty acid free BSA	0.25 mM ascorbic acid<br>2 ng/mL FGF7<br>0.25 μM SANT-1<br>0.1 μM retinoic acid<br>100 nM LDN193189<br>1:200 ITS-X<br>200 nM TPB
S5 (3 days)	3 Days	MCDB131<br>1.5 g/L Sodium bicarbonate<br>20 mM glucose<br>2% Fatty acid free BSA	0.25 μM SANT-1<br>0.05 μM retinoic acid<br>100 nM LDN193189<br>1:200 ITS-X<br>1 μM T3<br>10 μM ALK5i<br>10 μM Zinc Sulfate<br>10 μg/mL heparin
S6-1 (7 days)		MCDB131<br>1.5g/L Sodium bicarbonate<br>20mM glucose<br>2% Fatty acid free BSA	100 nM LDN193189<br>1:200 ITS-X<br>1 μM T3<br>10 μM ALK5i<br>10 μM Zinc Sulfate

			10 μg/mL heparin<br>100 nM GS inh XX
S6-2<br>(7<br>days)		MCDB131<br>1.5 g/L Sodium bicarbonate<br>20 mM glucose<br>2% Fatty acid free BSA	100 nM LDN193189<br>1:200 ITS-X<br>1 μM T3<br>10 μM ALK5i<br>10 μM Zinc Sulfate<br>10 μg/mL heparin<br>10 μM H1152

Supplementary Table 4. Related with Figure 2,4. Primer for qRT-PCR

Gene	Forward	Reverse
NKX6.1	CCGAGTCCTGCTTCTTCTTG	ATTCGTTGGGGATGACAGA G
MafA	GAGTTGGCACTTCTCGCTCT	TTCAGCAAGGAGGAGGTCA T
ABCC8	GAGAAGTCGGCCTCTTTGAA	GGGCCTTTGCCATCTATACC
UCN3	GATGGGCTTGGCTTTGTAGA	GGAGGGAAGTCCACTCTCG
G6PC2	ACACTCCAAAGAAATGACCAG G	CGCATCCTGTGTCTGGTATG
INS	GCAGCCTTTGTGAACCAACAC	CCCCGCACACTAGGTAGAG A
KCNK1	GGCCTTACCTCCATCTGACA	TGGAACTGGGACTTCACCTC
KCNK3	GAACATGCAGAACACCTTGC	GGCTCCTTCTACTTCGCCAT
PAX4	GAGGGTCTGGTTTTCCAACA	TGTGCAGAGATGATTCCTGG
SOX9	GTACCCGCACTTGCACAAC	GTGGTCCTTCTTGTGCTGC
NKX2.2	TCTACGACAGCAGCGACAAC	GGAGCTTGAGTCCTGAGGG
NEUROD 1	ATGACCAAATCGTACAGCGAG	GTTCATGGCTTCGAGGTCGT

Supplementary Table 5. Related with Figure 6. shRNA target sequences.

ROCKI	TCCATTCCATGGGTTTTAT
ROCKII	ATCAGACAGCATCCTTTCT
Scramble	GGACTACTCTAGACGTATA
